# The shift of obesity burden by socioeconomic status between 1998 and 2017 in Latin America and the Caribbean: a cross-sectional series study

**DOI:** 10.1016/S2214-109X(19)30421-8

**Published:** 2019-12-01

**Authors:** Safia S Jiwani, Rodrigo M Carrillo-Larco, Akram Hernández-Vásquez, Tonatiuh Barrientos-Gutiérrez, Ana Basto-Abreu, Laura Gutierrez, Vilma Irazola, Ramfis Nieto-Martínez, Bruno P Nunes, Diana C Parra, J Jaime Miranda

**Affiliations:** CRONICAS Center of Excellence in Chronic Diseases, Universidad Peruana Cayetano Heredia, Lima, Peru; Department of International Health, Johns Hopkins Bloomberg School of Public Health, Baltimore, MD, USA; CRONICAS Center of Excellence in Chronic Diseases, Universidad Peruana Cayetano Heredia, Lima, Peru; Department of Epidemiology and Biostatistics, School of Public Health, Imperial College London, London, UK; CRONICAS Center of Excellence in Chronic Diseases, Universidad Peruana Cayetano Heredia, Lima, Peru; Center for Population Health Research, National Institute of Public Health, Cuernavaca, Mexico; South American Center of Excellence for Cardiovascular Health, Institute for Clinical Effectiveness and Health Policy, Buenos Aires, Argentina; South Florida Veterans Affairs Foundation for Research and Education and Geriatric Research, Education and Clinical Center, Miami VA Healthcare System, Miami, FL, USA; Foundation for Clinical, Public Health, and Epidemiology Research in Venezuela, Caracas, Venezuela; Postgraduate Program of Nursing, Federal University of Pelotas, Rio Grande do Sul, Brazil; Program of Physical Therapy and Department of Surgery, Institute for Public Health, Washington University in St Louis School of Medicine, St Louis, MO, USA; CRONICAS Center of Excellence in Chronic Diseases, Universidad Peruana Cayetano Heredia, Lima, Peru; School of Medicine, Universidad Peruana Cayetano Heredia, Lima, Peru

## Abstract

**Background:**

The burden of obesity differs by socioeconomic status. We aimed to characterise the prevalence of obesity among adult men and women in Latin America and the Caribbean by socioeconomic measures and the shifting obesity burden over time.

**Methods:**

We did a cross-sectional series analysis of obesity prevalence by socioeconomic status by use of national health surveys done between 1998 and 2017 in 13 countries in Latin America and the Caribbean. We generated equiplots to display inequalities in, the primary outcome, obesity by wealth, education, and residence area. We measured obesity gaps as the difference in percentage points between the highest and lowest obesity prevalence within each socioeconomic measure, and described trends as well as changing patterns of the obesity burden over time.

**Findings:**

479 809 adult men and women were included in the analysis. Obesity prevalence across countries has increased over time, with distinct patterns emerging by wealth and education indices. In the most recent available surveys, obesity was most prevalent among women in Mexico in 2016, and the least prevalent among women in Haiti in 2016. The largest gap between the highest and lowest obesity estimates by wealth was observed in Honduras among women (21·6 percentage point gap), and in Peru among men (22·4 percentage point gap), compared with a 3·7 percentage point gap among women in Brazil and 3·3 percentage points among men in Argentina. Urban residents consistently had a larger burden than their rural counterparts in most countries, with obesity gaps ranging from 0·1 percentage points among women in Paraguay to 15·8 percentage points among men in Peru. The trend analysis done in five countries suggests a shifting of the obesity burden across socioeconomic groups and different patterns by gender. Obesity gaps by education in Mexico have reduced over time among women, but increased among men, whereas the gap has increased among women but remains relatively constant among men in Argentina.

**Interpretation:**

The increase in obesity prevalence in the Latin American and Caribbean region has been paralleled with an unequal distribution and a shifting burden across socioeconomic groups. Anticipation of the establishment of obesity among low socioeconomic groups could provide opportunities for societal gains in primordial prevention.

**Funding:**

None.

## Introduction

The characterisation of the association between obesity and elevated risks of chronic conditions, such as diabetes, heart disease, and some cancers, and all-cause mortality has been well researched.^1^ The prevalence of obesity has dramatically increased globally in the past two decades,^2^ owing to the nutrition transition and changes in dietary patterns, lifestyle, physical activity, and economic access.^3,4^

Although obesity has long been considered a condition of the elite and a mark of wealth, published literature in the past decade suggests that it can no longer be attributed to higher socioeconomic status.^4,5^ The burden of obesity is not static over time and the magnitude of such estimates are not necessarily the same across socioeconomic groups or across countries.^6^ A four-stage framework to approximate the epidemiology of obesity and its transition between socioeconomic status groups has been proposed on the basis of national data from 30 countries between 1975 and 2015.^7^ From a societal perspective, once the burden of obesity shifts to the most socioeconomically disadvantaged groups, it adds major challenges to other coexisting health and societal conditions and to the possibility of reverting to a non-obesity status. Such anticipation, or precision public health, requires an understanding of context and trends.

Latin America and the Caribbean has the largest income inequalities globally^8^ and has had an alarming increase in the prevalence of obesity since the 1990s, in parallel with rapidly growing urbanisation and economic growth.^4,9,10^ By use of data from Mexico, Brazil, and Colombia, one review^7^ suggests that Latin American countries are in stage 2 of the obesity transition, in which obesity prevalence has increased among the lower socioeconomic groups, and the gap by socioeconomic status groups has narrowed. The published literature on this issue needs to be updated, with the majority of studies covering a period in the early 2000s, and lacks information about obesity inequalities, particularly in the Latin American and Caribbean region.

We aimed to describe the obesity distribution and obesity gap by socioeconomic measures among adult men and women in 13 countries in Latin America and the Caribbean and evaluate the changing trend of obesity distribution and gap by socioeconomic measures over time in five countries with available data.

## Methods

### Study design and participants

We did a cross-sectional series analysis of obesity prevalence by socioeconomic status by use of national health surveys done in 13 countries in Latin America and the Caribbean. We used nationally representative health surveys done between 1998 and 2017 that included obesity and socioeconomic variables. Publicly available Demographic and Health Survey datasets were retrieved for Bolivia, Dominican Republic, Guatemala, Haiti, Honduras, Nicaragua, and Peru. Demographic and Health Surveys are nationally representative household surveys implemented in more than 90 low-income and middle-income countries that provide information on standard global health and population indicators. When Demographic and Health Surveys were not available, we used data from other nationally-representative health surveys: Argentina’s National Survey for Risk Factors, Brazil’s National Health Survey, Colombia’s National Survey on Nutritional Status, Mexico’s National Health and Nutrition Survey, Paraguay’s Non-communicable Disease Risk Factor survey, and the Venezuelan Cardiometabolic Health Study. Each survey had a distinct sampling design as outlined in the appendix 4 (pp 2–3).

The study population included individuals aged at least 18 years with available data on obesity. We excluded pregnant women from the analysis in all countries, except in Argentina where pregnancy status was not recorded in the dataset. Obesity data from Demographic and Health Surveys covered women aged 18–49 years, whereas data from other surveys covered women and men (if available) aged 18 years or older.

All surveys used for analysis included de-identified data. Ethical approval was not sought for this analysis of secondary data. All surveys except Brazil’s National Health Survey, Paraguay’s Non-communicable Disease Risk Factor survey, and the Venezuelan Cardiometabolic Health Study were publicly accessible.

### Procedures

For the analysis on obesity patterns and gaps by socioeconomic status, we did a cross-sectional analysis using the latest national health surveys in 13 countries: Argentina, Bolivia, Brazil, Colombia, Dominican Republic, Guatemala, Haiti, Honduras, Mexico, Nicaragua, Paraguay, Peru, and Venezuela. For the trend analysis on obesity gaps, we did a cross-sectional series analysis and included five countries (Argentina, Bolivia, Haiti, Mexico, and Peru) that had three consecutive surveys at least 4 years apart, with the most recent published after 2000.

The primary outcome, obesity, was defined as having a body-mass index (BMI) of 30 kg/m^2^ or more. Measured or reported weight and height variables were used to compute BMI. The three socioeconomic status measures of interest were wealth index (Q1–Q5, where Q1 is the poorest quintile and Q5 is the richest), education index (E1–E5, where E1 is the least educated quintile and E5 is the most educated), and area of residence (rural or urban). For countries with available Demographic and Health Surveys, we used the wealth quintiles existing in the datasets, generated by principal component analysis, which includes household ownership of assets, materials used for household construction, and access to water and sanitation facilities. Wealth index in Mexico was previously constructed using household characteristics (eg, number of rooms, exclusive kitchen, bathroom, and type of fuel) and household assets (eg, television, microwave, and computer), through principal component analysis; similarly, a wealth index based on a sum of asset ownership was developed in Brazil. For other national health surveys without existing wealth quintiles by principal component analysis, we computed wealth quintiles using alternative measures of wealth—eg, we used average monthly household income in Argentina and Paraguay. In all surveys, the wealth index was computed at the household level; therefore individuals residing in the same household belonged to the same wealth index category. The Venezuelan Cardiometabolic Health Study 2014–17 did not include any wealth variables; therefore we did not estimate obesity prevalence by wealth for this survey. The education index was generated into quintiles using the total number of formal years of education, as reported in Demographic and Health Surveys. When a continuous education variable was not available, we used an existing ascending categorical measure of education specified in the survey, such as in Argentina, Mexico, and Venezuela (no education and primary, secondary, and higher education). Area of residence was defined as rural or urban in all countries, except for Argentina’s National Survey for Risk Factors, which only sampled urban populations given that 91% of the Argentinian population reside in urban areas according to the 2010 census.^11^ In the case of Argentina, the obesity estimates computed therefore reflect the prevalence among urban populations. The data sources and socioeconomic status measure definitions used in each survey are summarised in the appendix 4 (pp 2–3).

### Statistical analysis

All 13 countries were included in the analysis of obesity gaps, whereas only five countries that had three consecutive surveys, at least 4 years apart, were included in the trend analysis of obesity gaps. We defined the gap in obesity prevalence as the absolute difference in percentage points between the highest and lowest most extreme obesity prevalence estimates within each socioeconomic status measure. For instance, if the highest obesity prevalence by wealth was observed among the third quintile, and the lowest among the fifth quintile, the obesity gap by wealth was calculated as the arithmetic difference between the obesity estimate in the third quintile and that in the fifth quintile. For the five countries with available data, we assessed the trends in obesity gaps by socioeconomic status over three timepoints. For the most recent surveys, we also reported the regional mean obesity prevalence within each socioeconomic status measure, computed as the arithmetic average of all countries’ estimate within each quintile.

We ran separate stratified analyses by gender for each country and survey. We calculated and reported the age-standardised obesity prevalence by each of the three socioeconomic status measures (wealth, education, and area of residence) using the WHO standard population age distribution.^12^

All analyses and graphs were conducted on Stata version 15. We used the *svy* command to account for complex survey sampling designs and the sampling weights for all countries’ surveys. We generated equiplots to display inequalities in obesity by socioeconomic status using the equiplot.ado file.

### Role of the funding source

There was no funding source for this study.

## Results

Data from 23 health surveys were used for this analysis, of which 13 were Demographic and Health Surveys. A total of 157 741 adult men and 322 068 adult women aged at least 18 years were included in the analysis: 282 247 men and women were included in analysis of the most recent obesity prevalence in the region, and 275 191 were included in the trend analysis of the change in prevalence over time.

The most recent data available for Latin America and the Caribbean corresponded to 2001–17, and the age-standardised obesity prevalence among adults varied greatly within the region (figure; tables 1, 2). Overall, the highest obesity prevalence was found among the fourth richest quintile (26·1%), third education quintile (27·1%), and urban (26·0%) women (table 1), whereas among men, the highest burden was in the richest quintile (24·5%), fourth education quintile (24·2%), and urban residents (22·0%; table 2). Mexico had the highest obesity prevalence by all three socioeconomic measures among men and women, whereas Haiti had the lowest obesity prevalence by wealth index among women and Colombia among men (table 1, 2).

The obesity prevalence varied by socioeconomic status measure and by country. Among women in Argentina, Brazil, Dominican Republic, Venezuela, and Paraguay, the burden was concentrated among the poor and least educated, particularly in Argentina in 2013, where 23·2% (95% CI 21·0–25·5) of the first wealth quintile and 28·5% (24·3–32·6) of the first education quintile were obese compared with 13·1% (10·4–15·7) of the fifth wealth quintile and 13·0% (11·6–14·3) of the fifth education quintile (table 1). Among men in Brazil, Colombia, and Paraguay, the richest and most educated quintiles had a higher obesity prevalence compared with lower wealth and education quintiles (table 2). The pattern was similar among women in Guatemala and Haiti and reversed among women in Colombia, where the obesity prevalence was lowest among the highest wealth and education quintiles (table 1).

Among women in Bolivia, Peru, Mexico, and Colombia, the prevalence was highest in the third or fourth wealth and third education quintiles. For example, among women in Mexico in 2016, the third wealth quintile had a 43·2% (95% CI 39·0–47·4) obesity prevalence compared with 37·2% (33·2–41·2) in the highest wealth quintile (table 1). Among men in Mexico, the prevalence was the highest in the fourth wealth and education quintiles (table 2).

In all countries except Venezuela, and Argentina where the comparison was not possible, the most recent surveys indicate that urban men had a higher obesity prevalence compared with their rural counterparts (table 2). This finding was consistent among women in Bolivia, Guatemala, Haiti, Honduras, Nicaragua, and Peru, and in the remaining countries albeit with overlapping CIs (table 1). The largest obesity prevalence was observed in Mexico in 2016, with 38·5% (95% CI 36·0–41·0) among urban women, 36·2% (33·4–39·0) among rural women, 28·6% (25·1–32·1) among urban men, and 21·8% (18·8–24·8) among rural men (tables 1, 2).

Multiple obesity patterns emerge by socioeconomic status (figure; table 1, 2): Bolivia, Guatemala, Haiti, Honduras, Peru, and Nicaragua had large inequalities in the distribution of obesity by wealth and education index; the widest obesity gaps among women were observed in Honduras, with a 21·6 percentage point difference in obesity prevalence by wealth, and Haiti, with a 20·4 percentage point difference, with the largest prevalence concentrated in the fourth wealth quintile in Honduras and the fifth in Haiti and the lowest prevalence among the poorest; and among men in Peru there was a 22·4 percentage point gap by wealth index between the highest prevalence among the fourth richest quintile and the lowest prevalence among the poorest quintile. Bolivia in 2008 had a bottom inequality pattern by wealth among women, in which large inequalities existed between the first and second poorest quintiles (9·0% [95% CI 7·5–10·4] *vs* 18·3% [16·4–20·2]), with smaller differences between subsequent quintiles (table 1).

In Colombia, Brazil, Mexico, and the Dominican Republic, the prevalence of obesity among women was similar in all wealth and education quintiles (table 1); this was also true for men in Argentina, where the gap in obesity prevalence between the first and second wealth quintiles was 3·3 percentage points (table 2). By area of residence, the largest obesity gaps were observed in Peru, with an 11·7 percentage point gap between urban and rural women and a 15·8 percentage point gap between urban and rural men (table 1, 2). The smallest obesity gap by area of residence was in Paraguay, with a 0·1 percentage point gap between urban and rural women (table 1).

The differential effect of socioeconomic status on obesity by gender was further confirmed in post-hoc analysis (appendix 4 p 4), in which gender modified the association between wealth index and obesity (in Argentina, Colombia, Paraguay, and Peru) and between education and obesity (in Colombia, Mexico, Paraguay, and Peru).

The trend analysis indicated that the prevalence and gap in obesity among women increased between 2005 and 2013 in Argentina (table 1; appendix 4 p 5): the obesity gap increased from 7·6 percentage points by wealth and 11·9 percentage points by education in 2005 to 10·2 percentage points and 15·5 percentage points in 2013. Although the obesity prevalence increased across all socioeconomic status groups among men between 2005 and 2013, the gap by socioeconomic status has remained relatively constant over time (appendix 4 p 6).

In 2016, Mexico had the highest obesity prevalence among men and women in the Latin American and Caribbean region, with women bearing a higher prevalence compared with men across socioeconomic status measures. Among women, the obesity prevalence has been increasing over time within each socioeconomic status measure (appendix 4 pp 7–8). The gap between the quintiles with the highest and lowest prevalence has increased slightly by wealth index among women (5·1 percentage points in 2006 compared with 8·3 percentage points in 2012 and 8·1 percentage points in 2016), although the prevalence remains highest among the third wealth quintile and lowest among the first (table 1; appendix 4 p 7). The gap has decreased by education and area of residence (4·4 percentage points in 2006 to 2·3 percentage points in 2016 by area of residence), with the highest prevalence remaining among the second and third education quintiles, and among urban residents (table 1; appendix 4 p 7). Among men, however, the obesity prevalence is different (appendix 4 p 8): in the three poorest quintiles, obesity has decreased between 2006 and 2016, and it has increased across all education quintiles in the same period, with the largest increase occurring in the first education quintile (18·2% in 2006 *vs* 28·2% in 2016; table 2). Similarly, obesity gaps have widened over time by wealth (9·5 percentage points *vs* 14·5 percentage points) and by education (8·2 percentage points *vs* 10·8 percentage points) between 2006 and 2016 but narrowed by residence (8·4 percentage points *vs* 6·8 percentage points; table 2; appendix 4 p 8).

In Peru, the prevalence of obesity among women increased in each group across all three socioeconomic status measures (appendix 4 p 9). The obesity gap by wealth reduced from 17·0 percentage points in 2005 to 13·4 percentage points in 2010, increasing to 16·0 percentage points in 2017, with substantial increases in the obesity prevalence among the poorest women (2·1% [95% CI 1·0–3·2] in 2005 to 16·4% [15·0–17·8] in 2017; table 1; appendix 4 p 9). However, the burden remains concentrated among the third and fourth wealth index quintiles. In terms of education, the gap in obesity prevalence between extreme quintiles has not varied substantially over time, but it has shifted; although the third education quintile retained the highest obesity prevalence between 2005 and 2017, the prevalence in the first quintile increased from 11·3% (95% CI 8·0–14·5) in 2005 to 25·1% (23·2–27·1) in 2017 (table 1; appendix 4 p 9). Both urban and rural women had an increasing obesity prevalence over time, with larger increases among urban residents (17·0% [95% CI 15·1–18·8] in 2005 to 30·1% [28·8–31·5] in 2017; table 1; appendix 4 p 9).

Patterns in obesity in Haiti differ greatly from the rest of the region between 2006 and 2016 (appendix 4 p 10): the rich, more educated, and urban women had the highest obesity prevalence. The prevalence among each wealth quintile and education quintile increased most between 2012 and 2016 (appendix 4 p 10). Although the overall obesity gap by wealth increased in magnitude between 2006 and 2016, it narrowed by education index from 13·3 percentage points to 11·3 percentage points, with the highest prevalence remaining among the richest groups, shifting from the fourth to the fifth education quintile, and the lowest prevalence remaining among the poorest and least educated (table 1; appendix 4 p 10). Additionally, the prevalence increased among both urban and rural women, shifting slightly towards rural residents, narrowing the obesity gap from 8·7 percentage points in 2006 to 7·9 percentage points in 2016 (table 1; appendix 4 p 10).

In Bolivia in 2008, the obesity prevalence was highest among the fourth wealth quintile, the third education quintile, and urban women, and it was lowest among the poorest, the most educated, and the rural residents (appendix 4 p 11). Between 1998 and 2008, the obesity gap widened by wealth (13·9 percentage points to 19·0 percentage points) and by education (7·9 percentage points to 12·3 percentage points), but narrowed by area of residence (9·4 percentage points to 7·5 percentage points), with larger increases in obesity prevalence among rural women (table 1; appendix 4 p 11).

## Discussion

Overall, our age-standardised obesity estimates suggest different obesity patterns across countries in the Latin American and Caribbean region, with the highest prevalence of obesity by socioeconomic status observed among women in Mexico in 2016 and the lowest among women in Haiti in 2016. We identified three distinct patterns for the distribution of obesity across socioeconomic status: concentration in the low wealth and education groups (Argentina, women in Venezuela, and women in Mexico by education), concentration in middle wealth and education groups (women in Bolivia, Peru, Mexico by wealth, and Colombia), and concentration among the high-income and high-education groups (women in Guatemala and Haiti and men in Mexico, Brazil, Colombia, Paraguay, Peru, and Venezuela). Moreover, the prevalence of obesity remains consistently higher among urban compared with rural men and women in most countries included in this analysis. However, with the exception of Peru, we found that increases in obesity have been larger among rural populations, which is in line with a global analysis^13^ showing that obesity among rural populations is increasing at a faster pace than that among urban populations. These patterns also suggest that countries in the Latin American and Caribbean region are in different stages in the transition of obesity as described by Jaacks and colleagues,^7^ according to socioeconomic groups and gender, thus tailoring of policies is required to adequately tackle the obesity epidemic in Latin America and the Caribbean.

In the early 2000s, obesity was believed to be a problem of the elite.^4,5^ However, evidence suggests a rapidly shifting prevalence towards lower socioeconomic groups, fueling inequalities in developing countries. This shift is believed to be associated with countries’ economic development,^14,15^ although the evidence remains unclear. Some studies in middle-income and high-income settings have suggested a reverse gradient, where the wealthier are more likely to be obese^3,5,16^ and where education protects against the obesogenic wealth effect,^17^ whereas other studies predict that the poor will eventually have a higher burden of chronic conditions, particularly in lower-income countries, where the prevalence of obesity seems to be shifting to the most disadvantaged groups as the country develops^14,15^ and the nutrition transition unfolds.^16,18^ The CARMELA study,^18^ a cross-sectional population-based observational study done in seven Latin American cities between April, 2004, and August, 2005, found an inverse relationship between socioeconomic status and obesity in adult women, particularly in the higher-income countries. Our results among women have now expanded this observation by indicating that, in lower-income settings, such as Haiti, Honduras, Nicaragua, and Guatemala, obesity is concentrated among the richer groups for women. However, in middle-income countries, such as Mexico, Colombia, Peru, and Brazil, the prevalence is highest in middle wealth groups among women and in wealthier, more educated groups among men.

The first pattern we observed, in which obesity is concentrated in the low education and wealth quintiles, is in line with a review of articles published between 1989 and 2003 by Monteiro and colleagues,^14,15^ which suggests that the prevalence of obesity was shifting more rapidly towards the lower socioeconomic status groups. Argentina, a country with very high human development index,^19^ fits this pattern. This result also fits Jaacks and colleagues^7^ obesity transition, with a reversal of the burden towards lower socioeconomic status groups (stage 3). However, the hypothetical stage 4 proposed by Jaacks and colleagues,^7^ in which obesity declines among all groups and the gap in obesity burden across socioeconomic groups narrows, was not observed in our study.

The second pattern was characterised by a high obesity prevalence in the third or fourth quintile for wealth, and in the third quintile for education, particularly in women. This pattern was observed in countries with high or medium human development index,^19^ such as Peru, Colombia, Mexico, and Bolivia. We hypothesise that these countries have entered the third stage of the obesity transition, whereby the prevalence of obesity is in the process of shifting towards the lower socio-economic status groups, possibly going through the middle socioeconomic status groups first. The two most recent surveys in Peru, Bolivia, and Mexico depict a similar situation of lowest obesity prevalence among the least socially advantaged women by wealth, as well as among the most socially advantaged women by education. This scenario confirms that the pathways by which socioeconomic indicators are associated with health outcomes differ depending on the indicator being used;^20^ therefore, wealth and education might be operating differently in the obesity epidemic, with the poorest and most educated women being shielded while those in the middle wealth and education groups have the highest prevalence.

The third pattern, characterised by a high prevalence of obesity among the high socioeconomic status groups, fits with the first stage of the obesity transition among women, whereby the burden is still concentrated among the higher socioeconomic status groups and has not yet shifted towards the lower socioeconomic status groups.^7^ This pattern was clearly observed among women in Guatemala, classified as medium in the human development index,^19^ and Haiti, classified as low. It was also found among men in Brazil, Colombia, Mexico, and Peru, which is in line with the proposed second stage of the obesity transition for men.^7^

Beyond differences by wealth and education, urban populations uniformly have a higher obesity prevalence compared with their rural counterparts, regardless of gender. However, the prevalence among rural residents has increased more rapidly than among urban residents, leading to narrower gaps in obesity prevalence between the two groups.^13,21–23^ A cross-sectional analysis of obesity prevalence among 147 938 non-pregnant women of reproductive age, using nationally representative data from between 1987 and 2000 in 38 countries, including nine in Latin America, indicated a scenario where obesity was equally distributed among the population in the Latin American countries.^9^ In contrast, an earlier analysis^4^ using survey data from between 1982 and 1996 showed that a third of obese women in the region came from poor rural areas, indicating a changing obesity burden, which is more in line with our results. Moreover, changes in policies in the past decade might also have affected the shifting burden of obesity in this region. Since 2006, 14 Latin American countries have adopted policies to reduce the consumption of sugar-sweetened beverages,^24^ including taxation in Mexico and Brazil.^25^ However, although the obesity epidemic is multifactorial, the effectiveness of such policies in reducing the obesity burden has not been well established,^25,26^ nor is a potential heterogenic effect across socioeconomic status well understood. Evidence suggests that such policies might be most effective in settings with high obesity prevalence and consumption of sugar-sweetened beverages.^25^

We also found that obesity prevalence in Latin America and the Caribbean appears to have distinct patterns by gender. With the exception of Argentina, the prevalence among men appears to be predominantly concentrated among the wealthier and the more educated groups, whereas this is not the case for women in the same countries. Among Argentinian men, the prevalence is concentrated among the third or fourth wealth quintiles, and shifts between the first and second education quintiles with the obesity gap remaining relatively constant between 2005 and 2013. Mexico is another example where women bear a larger prevalence of obesity compared with men: among women, we observed increasing trends and small, albeit increasing, obesity gaps by wealth, the prevalence being concentrated among middle-income groups, whereas men had a lower prevalence, concentrated among the richer and more educated, with larger obesity gaps. Our post-hoc statistical analyses confirmed that the association between socioeconomic status and obesity varies by gender. Beyond socioeconomic status, the differential effect of gender on obesity can be further explained by physiological and biological factors. Studies done in the USA, India, and China^27–30^ have reported a larger biological predisposition towards abdominal obesity and a higher prevalence of metabolic syndrome among women compared with men. In Peru and Brazil, studies have found a positive association between parity and BMI,^31,32^ and additional factors, such as environmental, genetic,^33^ hormonal, and non-hormonal, have also been suggested to differentially affect cardiovascular ageing mechanisms^34^ and metabolism^33^ between men and women.

Our study has several strengths, including the use of nationally representative surveys spanning a 20-year period, and could aid in informing more precise policy responses. It also has some limitations that stem from its cross-sectional design—ie, the trends we observed are based on estimates computed at specific timepoints and are not obtained from individual-level longitudinal data. Moreover, we compared obesity prevalence using the latest available health surveys, and the last survey for each country might cover a different period and sample size; this comparison is not ideal, and we ought to keep in mind contextual country-specific factors, such as differing periods of economic growth and development. Changes in obesity might not change drastically in the study periods, allowing a meaningful comparison across countries. Rather than making inferences comparing estimates between socioeconomic groups across countries, our analyses aim to descriptively show the changing distribution of obesity across socioeconomic status within countries. Similarly, we used different measures of wealth and education across countries, based on the variables collected, and we are not by any means comparing estimates in specific socioeconomic status groups between countries.^20,35^ In Argentina’s National Survey for Risk Factors, height and weight were self-reported by the respondent, whereas in all other surveys they were measured; hence obesity prevalence estimates for Argentina may bear recall-bias effects and lower accuracy. We did not compute absolute inequalities, instead we used equiplots to display the inequalities observed in the distribution of obesity across socioeconomic status and their directionality. Our sample included a much larger proportion of women than men, because the Demographic and Health Surveys mostly collect height and weight variables for women of reproductive age and children.

In conclusion, our analyses suggest great variability in the age-standardised obesity prevalence by wealth and education socioeconomic measures in Latin America and the Caribbean, whereas urban populations still maintain a larger prevalence than rural populations overall. Our findings also indicate that the prevalence of obesity is increasing in the region, with larger increases among rural residents and the most disadvantaged groups. However, the prevalence of obesity has been increasing not only among the poor, least educated, rural populations but also among the rich, highly educated, and urban populations. Among women, the obesity gap by wealth, education, and area of residence has stayed constant or widened in Argentina, Bolivia, Peru, and Mexico but has narrowed in Haiti by education and area of residence.

Ideally, a situation of low obesity prevalence within each socioeconomic status group and minimal obesity gaps would indicate that prevention and action should target the entire population. However, our analyses indicate that we are far from reaching this goal and that the obesity epidemic in Latin America and the Caribbean is complex, with distributions and trends varying across measures of socioeconomic status. In other words, wealth, education, or place of residence alone do not capture the full picture of the burden of obesity. To contain this epidemic and its heterogeneous spread, population-wide strategies are needed alongside programmes and policies that focus preventive interventions by socioeconomic status and by gender, advocating a more effective precision public health, rather than using a single approach. Adequate and frequent monitoring of the obesity epidemic is also needed in the region. Without updated data sources, countries will not be able to prioritise programmes and policies in the fight against obesity. Anticipation of the establishment of obesity among the low socioeconomic status groups offers opportunities for societal gains in primordial prevention. These findings can support efforts towards adequate monitoring of obesity by socioeconomic status groups that would allow anticipation of the transitions in obesity across societies and, thus, the formulation of tailored, equity-focused policy responses to the burden of obesity in the region.

## Supplementary Material

Supplementary Materials

## Figures and Tables

**Figure:**
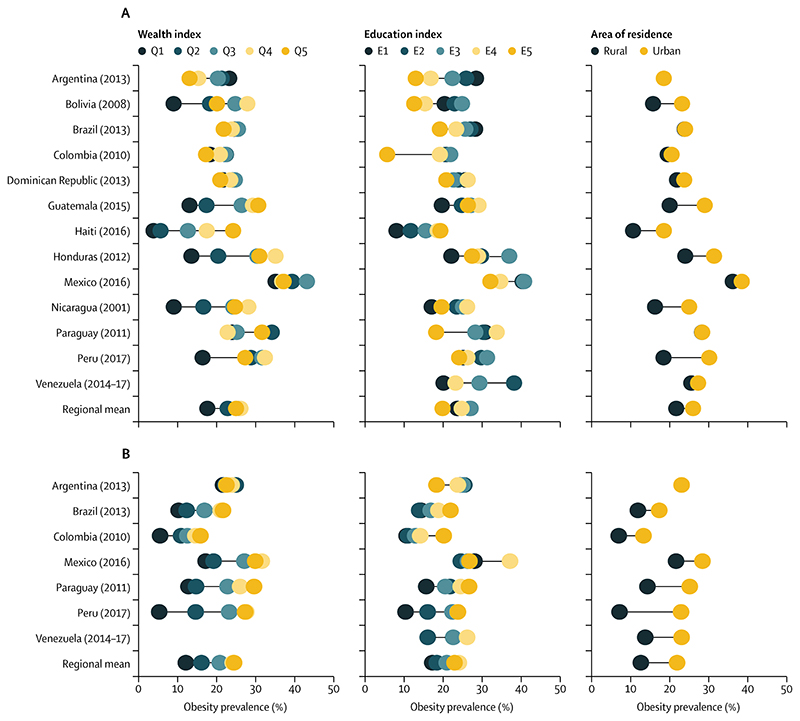
Most recent obesity prevalence by wealth, education, and residence (A) Among women. (B) Among men. Q=wealth quintile. E=education quintile.

**Table 1:** Age-standardised obesity prevalence among women by country and year

	Sample size, N*	Wealth index						Education index						Area of residence		
		Q1 (95% Cl)	Q2 (95% Cl)	Q3 (95% Cl)	Q4 (95% Cl)	Q5 (95% Cl)	Gap,† percentage points	E1 (95% Cl)	E2 (95% Cl)	E3 (95% Cl)	E4 (95% Cl)	E5 (95% Cl)	Gap,† percentage points	Rural (95% Cl)	Urban (95% Cl)	Gap,† percentage points
Argentina																
2005	21 037	18·2%	16·8%	16·0%	12·2%	10·6%	7·6	21·6%	16·9%	15·6%	11·8%	9·6%	11·9	..	13·8%	..
		(15·7–20·7)	(14·7–18·9)	(13·9–18·1)	(10·2–14·1)	(8·9–12·3)		(18·2–24·9)	(14·7–19·0)	(13·3–17·9)	(10·0–13·7)	(8·2–11·1)			(12·8–14·8)	
2009	18 095	22·2%	19·4%	15·8%	16·2%	13·4%	8·8	29·1%	23·0%	19·0%	14·7%	10·2%	18·9	..	16·8%	..
		(20·2–24·2)	(14·9–23·9)	(13·6–18·1)	(13·4–19·0)	(10·9–15·9)		(24·1–34·0)	(18·3–27·7)	(16·1–21·9)	(13·0–16·4)	(7·5–12·8)			(14·2–19·4)	
2013	16 664	23·2%	21·3%	20·3%	15·3%	13·1%	10·2	28·5%	25·9%	22·4%	16·8%	13·0%	15·5	..	18·5%	..
		(21·0–25·5)	(18·1–24·5)	(17·8–22·8)	(14·1–16·5)	(10·4–15·7)		(24·3–32·6)	(23·8–28·0)	(19·4–25·3)	(14·7–18·9)	(11·6–14·3)			(16·5–20·4)	
Bolivia																
1998	4125	5·5%	10·l%	15·9%	19·3%	14·4%	13·9	9·4%	17·3%	15·7%	13·9%	12·5%	7·9	6·6%	16·1%	9·4
		(3·6–7·3)	(7·5–12·6)	(12·9–19·0)	(15·2–23·5)	(10·9–18·0)		(7·2–11·6)	(14·0–20·7)	(12·1–19·3)	(10·6–17·1)	(9·3–15·7)		(5·1–8·2)	(13·6–18·5)	
2003	12 347	7·1%	14·9%	21·2%	22·5%	21·1%	15·5	15·2%	21·6%	24·2%	20·9%	13·5%	10·6	11·4%	21·5%	10·1
		(5·6–8·5)	(12·8–17·0)	(19·0–23·4)	(20·5–24·5)	(19·3–22·9)		(13·3–17·0)	(19·6–23·6)	(21·3–27·0)	(18·8–23·0)	(11·7–15·4)		(9·7–13·0)	(20·3–22·7)	
2008	13 497	9·0%	18·3%	24·8%	27·9%	20·1%	19·0	20·4%	22·9%	24·9%	15·3%	12·6%	12·3	15·7%	23·2%	7·5
		(7·5–10·4)	(16·4–20·2)	(22·8–26·8)	(25·9–29·9)	(18·3–21·9)		(18·6–22·1)	(20·8–25·1)	(23·2–26·6)	(10·1–20·6)	(10·9–14·2)		(14·3–17·1)	(22·1–24·3)	
Brazil, 2013	33 482	24·4%	25·4%	25·5%	23·9%	21·8%	3·7	28·2%	27·0%	25·7%	23·4%	19·2%	9·0	23·8%	24·0%	0·2
		(22·1–26·6)	(23·8–27·0)	(23·9–27·0)	(22·4–25·5)	(20·0–23·6)		(25·6–30·9)	(25·4–28·7)	(23·8–27·6)	(21·9–24·8)	(17·2–21·1)		(22·1–25·6)	(23·1–24·8)	
Colombia, 2010	47 272	18·3%	22·4%	22·2%	20·9%	17·3%	5·1	20·8%	20·7%	21·8%	19·2%	5·6%	16·2	19·5%	20·5%	1·0
		(17·3–19·4)	(21·2–23·6)	(21·1–23·4)	(19·6–22·1)	(16·1–18·5)		(19·9–21·8)	(19·7–21·7)	(20·1–23·6)	(18·2–20·1)	(5·4–5·8)		(18·4–20·5)	(19·8–21·1)	
Dominican Republic, 2013	7655	21·6%	24·0%	24·7%	23·5%	20·9%	3·8	25·7%	23·9%	22·7%	26·3%	20·8%	5·5	21·9%	23·7%	1·9
		(19·0–24·1)	(21·1–27·0)	(22·1–27·3)	(20·8–26·2)	(18·5–23·4)		(23·5–27·9)	(20·9–26·9)	(20·3–25·1)	(21·1–31·6)	(18·2–23·4)		(19·7–24·1)	(22·3–25·2)	
Guatemala, 2015	14 256	13·0%	17·4%	26·4%	29·3%	30·7%	17·7	19·9%	24·9%	27·1%	29·1%	26·4%	9·2	20·0%	29·0%	9·0
		(11·3–14·8)	(15·6–19·2)	(24·4–28·4)	(27·2–31·4)	(28·7–32·7)		(18·1–21·6)	(22·8–27·0)	(24·7–29·5)	(26·9–31·3)	(23·8–28·9)		(18·6–21·3)	(27·5–30·5)	
Haiti																
2006	2528	0·7%	2·4%	6·6%	11·3%	19·2%	18·5	5·0%	6·6%	10·1%	18·4%	16·6%	13·3	6·0%	14·7%	8·7
		(-0·2–1·5)	(0·6–4·1)	(3·7–9·6)	(7·9–14·7)	(15·1–23·4)		(2·8–7·2)	(4·2–9·1)	(6·4–13·7)	(13·9–22·8)	(13·3–19·9)		(3·9–8·0)	(11·6–17·7)	
2012	7462	1·5%	4·9%	6·7%	12·l%	19·3%	17·8	4·9%	9·2%	12·8%	16·5%	15·4%	11·7	7·0%	14·2%	7·2
		(0·8–2·2)	(3·4–6·3)	(5·1–8·4)	(10·0–14·2)	(16·7–21·9)		(3·7–6·1)	(7·4–11·0)	(9·7–15·9)	(13·4–19·7)	(12·5–18·4)		(5·7–8·3)	(12·4–16·0)	
2016	7667	3·8%	5·6%	12·6%	17·5%	24·2%	20·4	8·0%	11·7%	15·5%	18·6%	19·3%	11·3	10·6%	18·5%	7·9
		(2·6–5·0)	(4·4–6·8)	(10·6–14·7)	(15·4–19·6)	(21·6–26·9)		(5·9–10·1)	(9·8–13·6)	(13·0–18·1)	(15·5–21·7)	(16·3–22·2)		(9·1–12·0)	(16·7–20·3)	
Honduras, 2012	10 061	13·5%	20·4%	30·5%	35·1%	31·0%	21·6	22·1%	29·7%	37·0%	29·0%	27·4%	14·9	24·0%	31·4%	7·4
		(11·7–15·4)	(18·4–22·5)	(28·0–32·9)	(32·0–38·3)	(28·3–33·6)		(19·9–24·2)	(27·9–31·6)	(29·7–44·2)	(26·3–31·6)	(23·8–31·0)		(21·9–26·0)	(29·6–33·2)	
Mexico																
2006	21 334	32·2%	34·1%	36·2%	31·4%	31·1%	5·1	30·9%	36·3%	35·9%	29·7%	25·3%	11·0	29·8%	34·2%	4·4
		(30·6–33·8)	(32·2–36·1)	(34·0–38·4)	(28·8–34·0)	(27·6–34·7)		(27·6–34·2)	(34·7–37·9)	(33·6–38·2)	(27·3–32·2)	(22·5–28·1)		(27·8–31·8)	(32·9–35·5)	
2012	23 707	30·7%	37·5%	39·0%	39·0%	35·3%	8·3	33·4%	39·7%	38·6%	32·3%	30·8%	8·9	32·7%	37·2%	4·4
		(28·8–32·6)	(35·6–39·5)	(37·1–40·8)	(36·8–41·2)	(33·2–37·3)		(29·9–36·9)	(38·0–41·4)	(36·6–40·5)	(30·1–34·6)	(28·2–33·4)		(31·1–34·2)	(36·0–38·4)	
2016	5889	35·1%	39·4%	43·2%	36·8%	37·2%	8·1	40·4%	40·6%	40·8%	34·7%	32·1%	8·7	36·2%	38·5%	2·3
		(31·1–39·1)	(35·8–42·9)	(39·0–47·4)	(33·6–40·1)	(33·2–41·2)		(35·4–45·4)	(37·4–43·8)	(37·4–44·3)	(30·8–38·6)	(28·1–36·2)		(33·4–39·0)	(36·0–41·0)	
Nicaragua, 2001	10 186	9·0%	16·6%	24·3%	28·2%	24·7%	19·2	17·0%	23·5%	25·1%	26·2%	19·6%	9·1	16·2%	25·0%	8·8
		(7·5–10·5)	(14·6–18·6)	(22·0–26·5)	(26·0–30·4)	(22·0–27·4)		(15·0–19·0)	(21·3–25·6)	(22·8–27·4)	(23·1–29·3)	(l7·2–22·0)		(14·6–17·9)	(23·4–26·5)	
Paraguay, 2011	1509	24·0%	34·2%	25·1%	22·8%	31·7%	11·4	30·8%	30·3%	28·3%	33·8%	18·2%	15·6	28·3%	28·3%	0·1
		(19·7–28·4)	(29·9–38·5)	(20·1–30·2)	(18·3–27·3)	(26·1–37·2)		(26·8–34·7)	(26·1–34·6)	(24·2–32·4)	(28·7–38·8)	(14·3–22·1)		(24·9–31·7)	(25·5–31·2)	
Peru																
2005	4911	2·1%	10·7%	19·1%	17·6%	15·7%	17·0	11·3%	18·1%	18·4%	13·4%	12·3%	7·1	8·6%	17·0%	8·4
		(1·0–3·2)	(7·9–13·5)	(15·7–22·5)	(14·5–20·6)	(13·1–18·4)		(8·0–14·5)	(14·4–21·7)	(15·6–21·1)	(8·9–17·9)	(10·1–14·5)		(5·7–11·5)	(15·1–18·8)	
2010	18 837	8·5%	15·2%	21·1%	21·8%	17·9%	13·4	16·5%	20·4%	20·6%	15·0%	14·6%	6·0	10·8%	19·9%	9·1
		(7·4–9·5)	(13·8–16·7)	(19·5–22·7)	(20·0–23·6)	(16·1–19·8)		(14·6–18·4)	(18·6–22·1)	(18·9–22·2)	(11·8–18·2)	(13·0–16·2)		(9·9–11·8)	(18·9–20·9)	
2017	17 210	16·4%	28·8%	31·7	32·4%	27·3%	16·0	25·1%	29·8%	31·3%	26·2%	24·1%	7·2	18·4%	30·1%	11·7
		(15·0–17·8)	(27·0–30·7)	(29·5–33·9)	(30·1–34·8)	(24·7–30·0)		(23·2–27·1)	(27·4–32·1)	(29·0–33·6)	(23·8–28·7)	(21·5–26·7)		(17·1–19·8)	(28·8–31·5)	
Venezuela, 2014–17	2337	..	..	..	..	..	..	20·1%	38·2%	29·3%	23·2%	NA	18·1	25·5%	27·3%	1·8
								(18·9–21·3)	(32·8–43·6)	(23·5–35·1)	(17·8–28·5)			(19·0–32·1)	(23·2–31·4)	
Regional mean‡	..	17·6%	22·8%	25·9%	26·1%	25·0%	8·5	23·6%	26·8%	27·1%	24·7%	19·8%	7·3	21·7%	26·0%	4·3

NA=not applicable.

* Unweighted.

† Gap defined as the difference between the highest and lowest obesity prevalence within the socioeconomic status measure.

‡ Regional mean is the arithmetic average of country estimates for the most recent surveys.

**Table 2:** Age-standardised obesity prevalence among men by country and year

	Sample size, N*	Wealth index						Education index						Area of residence		
		Q1 (95% Cl)	Q2 (95% Cl)	Q3 (95% Cl)	Q4 (95% Cl)	Q5 (95% Cl)	Gap,† percentage points	E1 (95% Cl)	E2 (95% Cl)	E3 (95% Cl)	E4 (95% Cl)	E5 (95% Cl)	Gap,† percentage points	Rural (95% Cl)	Urban (95% Cl)	Gap,† percentage points
Argentina																
2005	16 918	15·0%	16·5%	15·3%	17·7%	14·5%	3·2	19·8%	17·2%	17·8%	15·2%	12·3%	7·5	..	15·6%	..
		(12·5–17·5)	(13·9–19·0)	(13·3–17·3)	(15·2–20·2)	(12·7–16·4)		(16·8–22·7)	(15·0–19·5)	(15·4–20·3)	(13·1–17·5)	(10·6–13·9)			(14·4–16·7)	
2009	14 353	17·3%	21·1%	21·2%	20·4%	15·8%	5·4	19·1%	20·2%	22·3%	19·1%	15·6%	6·7	..	19·3%	..
		(14·8–19·8)	(19·4–22·9)	(18·1–24·3)	(18·6–22·2)	(13·4–18·1)		(16·5–21·6)	(18·3–22·2)	(19·2–25·4)	(18·0–20·3)	(14·3–17·0)			(18·3–20·3)	
2013	13 626	21·7%	25·0%	22·4%	23·9%	22·6%	3·3	24·4%	25·6%	25·1%	23·9%	18·4%	7·2	..	23·2%	..
		(19·6–23·8)	(21·9–28·0)	(20·1–24·7)	(20·7–27·1)	(20·0–25·1)		(20·3–28·6)	(23·8–27·4)	(22·0–28·2)	(21·7–26·2)	(15·9–20·9)			(21·7–24·7)	
Brazil, 2013	25 920	10·2%	12·3%	16·9%	21·1%	21·7%	11·5	14·5%	14·0%	17·0%	18·9%	22·0%	8·0	11·9%	17·5%	5·6
		(8·8–11·6)	(11·0–13·6)	(15·3–18·5)	(19·0–23·1)	(19·8–23·5)		(12·5–16·4)	(12·8–15·2)	(15·1–18·8)	(17·4–20·5)	(20·1–24·0)		(10·5–13·4)	(16·5–18·4)	
Colombia, 2010	36 544	5·5%	10·9%	12·5%	14·6%	15·9%	10·4	10·7%	11·1%	13·0%	14·3%	20·3% (NA)	9·6	7·0%	13·4%	6·4
		(4·8–6·2)	(9·9–11·8)	(11·5–13·5)	(13·4–15·8)	(14·6–17·2)		(9·8–11·6)	(10·2–12·0)	(11·4–14·5)	(13·3–15·2)			(6·3–7·8)	(12·8–14·0)	
Mexico																
2006	14 394	19·7%	21·4%	29·2%	28·0%	25·6%	9·5	18·2%	23·8%	26·4%	25·7%	25·6%	8·2	17·1%	25·4%	8·4
		(18·1–21·4)	(18·9–23·2)	(26·8–31·6)	(25·2–30·9)	(22·0–29·3)		(15·1–21·3)	(21·8–25·7)	(24·1–28·7)	(22·7–28·6)	(22·7–28·4)		(15·0–19·1)	(24·1–26·8)	
2012	17 514	17·4%	24·6%	26·5%	28·8%	30·4%	13·0	19·3%	23·5%	27·7%	29·l%	29·8%	10·6	19·7%	28·1%	8·4
		(15·7–19·1)	(22·6–26·6)	(24·5–28·6)	(26·4–31·2)	(28·1–32·7)		(15·9–22·7)	(21·9–25·2)	(25·6–29·7)	(26·5–31·6)	(27·2–32·5)		(18·3–21·1)	(26·8–29·4)	
2016	3076	17·2%	19·2%	27·2%	31·7%	30·0%	14·5	28·2%	24·7%	26·5%	37·4%	26·9%	10·8	21·8%	28·6%	6·8
		(13·8–20·6)	(16·4–22·1)	(23·2–31·2)	(27·3–36·0)	(24·9–35·0)		(22·5–34·0)	(20·8–28·5)	(23·1–30·0)	(33·1–41·6)	(22·1–31·8)		(18·8–24·8)	(25·1–32·1)	
Paraguay, 2011	876	12·8%	14·7%	22·9%	26·1%	29·7%	16·9	15·8%	21·9%	20·7%	24·7%	26·8%	11·0	14·4%	25·3%	10·9
		(9·4–16·2)	(10·7–18·8)	(18·4–27·3)	(19·9–32·3)	(24·6–34·8)		(11·6–19·9)	(16·6–27·3)	(16·0–25·4)	(20·1–29·3)	(21·9–31·7)		(10·6–18·2)	(21·8–28·8)	
Peru 2017	13 466	5·3%	14·6%	23·3%	27·7%	27·4	22·4	10·5%	16·2%	22·6%	23·9%	24·0	13·5	7·2	23·1%	15·8
		(4·4–61)	(131·16–2)	(20·9–25·7)	(24·7–30·6)	(24·6–30·1)		(8·2–12·7)	(13·9–18·4)	(20·5–24·6)	(21·6–26·1)	(21·0–26·9)		(6·2–8·2)	(21·7–24·4)	
Venezuela, 2014–17	1054	..	..	..	..	..	..	..	16·2%	22·7%	26·3%	NA	10·2	13·8%	23·2%	9·4
									(13·9–18·4)	(16·1–29·4)	(16·6–36·1)			(9·2–18·4)	(15·8–30·6)	
Regional mean‡	..	12·1%	16·1%	20·9%	24·2%	24·5%	12·4	17·4%	18·5%	21·1%	24·2%	23·1%	6·8	12·7%	22·0%	9·3

NA=not applicable.

* Unweighted.

† Gap defined as the difference between the highest and lowest obesity prevalence within the socioeconomic status measure.

‡ Regional mean is the arithmetic average of country estimates for the most recent surveys.

## Research in context

### Evidence before this study

Obesity has long been believed to affect the elite; however, research in the past decade suggests a rapid shift of the burden of obesity towards lower socioeconomic groups. The prevalence of obesity has been increasing in countries of the Latin America and Caribbean region since the 1990s. We did a PubMed literature search for articles on adult obesity prevalence and trends in Latin America and the Caribbean published between Jan 1, 2010, and April 1, 2019, with “adult obesity” and “Latin America” or “South America” or “Carribean” in the title. On the basis of title review we identified few articles that matched our search criteria: the majority of articles were clinical or experimental in nature. Jaacks and colleagues explored the epidemiology of obesity between 1975 and 2015 in 30 countries, representing more than 75% of the world’s population, including Mexico, Colombia, and Brazil. A four-stage obesity transition model was proposed, in which the obesity burden concentrated among women and higher socioeconomic status groups (particularly for women; stage 1) shifts towards the more disadvantaged, narrowing the gap between sexes and between socioeconomic status groups among women (stage 2), until a reversal of the burden occurs where obesity prevalence among lower socioeconomic status surpasses that of the higher socioeconomic status groups (stage 3), after which declines in obesity would be expected (stage 4). Nevertheless, given that only three countries of the Latin American and Caribbean region were represented in this study, the evidence on the differential burden of obesity between high and low socioeconomic groups in this region remains unclear.

### Added value of this study

The published literature on this issue remains outdated with the majority of studies covering a period in the early 2000s. In this analysis, we provide an update on the current distribution of the obesity burden across socioeconomic status in Latin America and the Caribbean and the changing burden over time. In particular, our findings point to the shifting patterns of the obesity burden across gender and socioeconomic status in the Latin American and Caribbean region. Our findings serve as a call to action for tailored, equity-focused programmes and policies.

### Implications of all the available evidence

Latin America and the Caribbean is the region with the largest income inequalities globally. These inequalities, coupled with rapid urbanisation and economic growth, increase the risk of growing obesity rates. Therefore, up-to-date information on the magnitude of the problem by various socioeconomic measures can help guide and target prevention efforts.

## References

[1] Di Angelantonio E, Bhupathiraju S, Global BMI Mortality Collaboration (2016). Body-mass index and all-cause mortality: individual-participant-data meta-analysis of 239 prospective studies in four continents. Lancet.

[2] NCD Risk Factor Collaboration (2016). Trends in adult body-mass index in 200 countries from 1975 to 2014: a pooled analysis of 1698 population-based measurement studies with 19.2 million participants. Lancet.

[3] Jeon H, Salinas D, Baker DP (2015). Non-linear education gradient across the nutrition transition: mothers’ overweight and the population education transition. Public Health Nutr.

[4] Martorell R, Khan LK, Hughes ML, Grummer-Strawn LM (1998). Obesity in Latin American women and children. J Nutr.

[5] Poterico JA, Stanojevic S, Ruiz-Grosso P, Bernabe-Ortiz A, Miranda JJ (2012). The association between socioeconomic status and obesity in Peruvian women. Obesity.

[6] Carrillo-Larco RM, Miranda JJ, Bernabé-Ortiz A (2016). Wealth index and risk of childhood overweight and obesity: evidence from four prospective cohorts in Peru and Vietnam. Int J Public Health.

[7] Jaacks LM, Vandevijvere S, Pan A (2019). The obesity transition: stages of the global epidemic. Lancet Diabetes Endocrinol.

[8] Dansereau E, McNellan CR, Gagnier MC (2016). Coverage and timing of antenatal care among poor women in 6 Mesoamerican countries. BMC Pregnancy Childbirth.

[9] Martorell R, Khan LK, Hughes ML, Grummer-Strawn LM (2000). Obesity in women from developing countries. Eur J Clin Nutr.

[10] Uauy R, Albala C, Kain J (2001). Obesity trends in Latin America: transiting from under-to overweight. J Nutr.

[11] Ministerio de Defensa Población de la República Argentina.

[12] Ahmad OB, Boschi-Pinto C, Lopez AD, Murray CJL, Lozano R, Inoue M (2001). Age standardization of rates: a new WHO standard.

[13] NCD Risk Factor Collaboration (2019). Rising rural body-mass index is the main driver of the global obesity epidemic in adults. Nature.

[14] Monteiro CA, Conde WL, Lu B, Popkin BM (2004). Obesity and inequities in health in the developing world. Int J Obes Relat Metab Disord.

[15] Monteiro CA, Moura EC, Conde WL, Popkin BM (2004). Socioeconomic status and obesity in adult populations of developing countries: a review. Bull World Health Organ.

[16] Jones-Smith JC, Gordon-Larsen P, Siddiqi A, Popkin BM (2012). Is the burden of overweight shifting to the poor across the globe? Time trends among women in 39 low-and middle-income countries (1991-2008). Int J Obes.

[17] Aitsi-Selmi A, Bell R, Shipley MJ, Marmot MG (2014). Education modifies the association of wealth with obesity in women in middle-income but not low-income countries: an interaction study using seven national datasets, 2005-2010. PLoS One.

[18] Boissonnet C, Schargrodsky H, Pellegrini F (2011). Educational inequalities in obesity, abdominal obesity, and metabolic syndrome in seven Latin American cities: the CARMELA study. Eur J Cardiovasc Prev Rehabil.

[19] United Nations Development Programme (2018). Human development indices and indicators: 2018 statistical update.

[20] Howe LD, Galobardes B, Matijasevich A (2012). Measuring socio-economic position for epidemiological studies in low-and middle-income countries: a methods of measurement in epidemiology paper. Int J Epidemiol.

[21] Parra DC, Gomez LF, Iannotti L, Haire-Joshu D, Sebert Kuhlmann AK, Brownson RC (2018). Maternal and familial correlates of anthropometric typologies in the nutrition transition of Colombia, 2000-2010. Public Health Nutr.

[22] Carrillo-Larco RM, Miranda JJ, Gilman RH (2018). Trajectories of body mass index and waist circumference in four Peruvian settings at different level of urbanisation: the CRONICAS cohort study. J Epidemiol Community Health.

[23] Parra DC, Iannotti L, Gomez LF (2015). The nutrition transition in Colombia over a decade: a novel household classification system of anthropometric measures. Arch Public Health.

[24] Bergallo P, Castagnari V, Fernández A, Mejía R (2018). Regulatory initiatives to reduce sugar-sweetened beverages (SSBs) in Latin America. PLoS One.

[25] Jou J, Techakehakij W (2012). International application of sugar-sweetened beverage (SSB) taxation in obesity reduction: factors that may influence policy effectiveness in country-specific contexts. Health Policy.

[26] Fernandez MA, Raine KD (2019). Insights on the influence of sugar taxes on obesity prevention efforts. Curr Nutr Rep.

[27] Mauvais-Jarvis F (2017). Epidemiology of gender differences in diabetes and obesity. Adv Exp Med Biol.

[28] Aguilar M, Bhuket T, Torres S, Liu B, Wong RJ (2015). Prevalence of the metabolic syndrome in the United States, 2003-2012. JAMA.

[29] Gu D, Reynolds K, Wu X (2005). Prevalence of the metabolic syndrome and overweight among adults in China. Lancet.

[30] Gupta R, Deedwania PC, Gupta A, Rastogi S, Panwar RB, Kothari K (2004). Prevalence of metabolic syndrome in an Indian urban population. Int J Cardiol.

[31] Huayanay-Espinoza CA, Quispe R, Poterico JA, Carrillo-Larco RM, Bazo-Alvarez JC, Miranda JJ (2017). Parity and overweight/obesity in Peruvian women. Prev Chronic Dis.

[32] Reis-Santos B, Barros FC, Horta BL (2018). Is there a causal effect of parity on body composition: a birth cohort study. BMC Public Health.

[33] Link JC, Reue K (2017). Genetic basis for sex differences in obesity and lipid metabolism. Annu Rev Nutr.

[34] Merz AA, Cheng S (2016). Sex differences in cardiovascular ageing. Heart.

[35] McCartney G, Bartley M, Dundas R (2018). Theorising social class and its application to the study of health inequalities. SSM Popul Health.

