# Supplementary material for: The shift of obesity burden by socioeconomic status between 1998 and 2017 in Latin America and the Caribbean: a cross-sectional series study
Source: Lancet Glob Health. Author manuscript; Available in PMC 2022 Jul 17. (PMC7613084; doi:10.1016/S2214-109X(19)30421-8)

THE LANCET
Global Health

Supplementary appendix 4

This appendix formed part of the original submission and has been peer reviewed. We post it as supplied by the authors.

This online publication has been corrected. The corrected version first appeared at thelancet.com/lancetgh on Jan 24, 2020

Supplement to: Jiwani SS, Carrillo-Larco RM, Hernández-Vásquez A, et al. The shift of obesity burden by socioeconomic status between 1998 and 2017 in Latin America and the Caribbean: a cross-sectional series study. *Lancet Glob Health* 2019; **7:** e1644–54.

The shift of obesity burden by socioeconomic status between 1998 and 2017 in Latin America and the Caribbean: A cross-sectional series study

APPENDIX

Supplementary table 1A. Indicator definition by country

Country	Survey(s)	Survey Design	BMI measure	Wealth Index (Q1-Q5)	Education Index (E1-E5)	Area of Residence
Argentina	ENFR 2005<br>ENFR 2009<br>ENFR 2013	Stratified four-stage cluster design	Reported height and weight	Wealth quintiles based on average monthly household income	1. No education/ primary incomplete<br>2. Primary complete<br>3. Secondary incomplete<br>4. Secondary complete<br>5. Higher/special education	Urban only
Bolivia	DHS<br>1998<br>2003<br>2008	Stratified two-stage cluster design	Measured height and weight	Wealth quintiles based on principal component analysis	Education quintiles based on number of years of education	Urban/Rural
Brazil	PNS 2013	Stratified three-stage cluster design	Measured height and weight	Wealth quintiles based on sum of assets ownership	1. No education<br>2. Primary incomplete<br>3. Primary complete and secondary incomplete<br>4. Secondary complete and higher incomplete<br>5. Higher education complete	Urban/Rural
Colombia	ENSIN 2010	Three-stage cluster design	Measured height and weight	Wealth quintiles based on principal component analysis	Education quintiles based on number of years of education	Urban/Rural
Dominican Republic	DHS 2013	Stratified two-stage cluster design	Measured height and weight	Wealth quintiles based on principal component analysis	Education quintiles based on number of years of education	Urban/Rural
Guatemala	DHS 2015	Stratified two-stage cluster design	Measured height and weight	Wealth quintiles based on principal component analysis	Education quintiles based on number of years of education	Urban/Rural
Haiti	DHS<br>2000<br>2006<br>2016	Stratified two-stage cluster design	Measured height and weight	Wealth quintiles based on principal component analysis	Education quintiles based on number of years of education	Urban/Rural
Honduras	DHS 2012	Stratified two-stage cluster design	Measured height and weight	Wealth quintiles based on principal component analysis	Education quintiles based on number of years of education	Urban/Rural

Mexico	ENSANUT 2006 2012 2016	Stratified three-stage cluster design	Measured height and weight	Wealth quintiles based on principal component analysis	1. Pre-school or less (<6 years)<br>2. Elementary school (6 years)<br>3. Middle school (9 years)<br>4. High school (12 years)<br>5. Higher education (>12 years)	Urban/Rural
Nicaragua	DHS 2001	Stratified two-stage cluster design	Measured height and weight	Wealth quintiles based on principal component analysis	Education quintiles based on number of years of education	Urban/Rural
Paraguay	STEP 2011	Three-stage cluster design	Measured height and weight	Wealth quintiles based on average monthly household income	Education quintiles based on number of years of education	Urban/Rural
Peru	ENDES 2005 2010 2017	Stratified two-stage cluster design	Measured height and weight	Wealth quintiles based on principal component analysis	Education quintiles based on number of years of education	Urban/Rural
Venezuela	EVESCAM 2014-2017	Stratified three-stage cluster design	Measured height and weight	N/A	1. No education<br>2. Primary (incomplete & complete)<br>3. Secondary (incomplete & complete)<br>4. University (and higher)	Urban/Rural

Supplementary table 2A. Differential effect of socioeconomic status on obesity by gender

	Argentina ENFR 2013			Brazil PNS 2013			Colombia ENSIN 2010			Mexico ENSANUT 2016			Paraguay STEP 2011			Peru ENDES 2017		
	PR	95% CI	p value	PR	95% CI	p value	PR	95% CI	p value	PR	95% CI	p value	PR	95% CI	p value	PR	95% CI	p value
Interaction: Wealth Index * Gender																		
Q1*Female (ref)																		
Q2*Female	0.78	0.64-0.95	**0.02**	0.84	0.68-1.04	0.10	0.61	0.52-0.72	**<0.01**	1.00	0.66-1.51	0.99	0.69	0.45-2.02	0.91	0.62	0.50-0.78	**<0.01**
Q3*Female	0.88	0.72-1.08	0.19	0.64	0.52-0.79	**<0.01**	0.54	0.46-0.64	**<0.01**	0.77	0.52-1.15	0.20	0.67	0.60-1.49	0.33	0.44	0.35-0.54	**<0.01**
Q4*Female	0.61	0.49-0.77	**<0.01**	0.53	0.44-0.64	**<0.01**	0.43	0.37-0.51	**<0.01**	0.65	0.42-1.00	0.05	0.41	0.19-0.88	**0.02**	0.39	0.31-0.48	**<0.01**
Q5*Female	0.57	0.46-0.70	**<0.01**	0.43	0.35-0.52	**<0.01**	0.35	030-0.41	**<0.01**	0.64	0.42-0.99	0.42	0.50	0.25-1.01	0.05	0.35	0.28-0.44	**<0.01**
Adjusted Wald Test			**<0.01**			**<0.01**			**<0.01**			0.11			**0.02**			**<0.01**
Interaction: Education Index * Gender																		
E1*Female (ref)																		
E2*Female	0.93	0.72-1.19	0.50	.81	0.67-0.97	**0.02**	0.99	0.87-1.13	0.86	0.74	0.47-1.17	0.20	0.69	0.43-1.11	0.13	0.79	0.61-1.03	0.08
E3*Female	0.89	0.62-1.28	0.48	.65	0.56-0.75	**<0.01**	0.92	0.77-1.10	0.34	0.83	0.50-1.38	0.48	0.72	0.43-1.19	0.20	0.52	0.41-0.66	**<0.01**
E4*Female	0.68	0.48-0.96	**0.03**	.45	0.38-0.54	**<0.01**	0.72	0.64-0.82	**<0.01**	0.44	0.27-0.72	**<0.01**	0.68	0.42-1.11	0.12	0.42	0.33-0.54	**<0.01**
E5*Female	0.70	0.48-1.01	0.05	-	-	-	0.32	0.13-0.76	**0.01**	0.48	0.24-0.94	**0.03**	0.43	0.26-0.72	**<0.01**	0.38	0.29-0.49	**<0.01**
Adjusted Wald Test			0.20			**<0.01**			**<0.01**			**<0.01**			**0.03**			**<0.01**

Wealth Index defined as Q1 being poorest quintile, and Q5 being the richest; Education Index defined as E1 being the least educated, and E5 being the most educated

Supplementary figure 1A. Trend in age-standardized obesity prevalence by wealth, education and residence among adult women in Argentina*

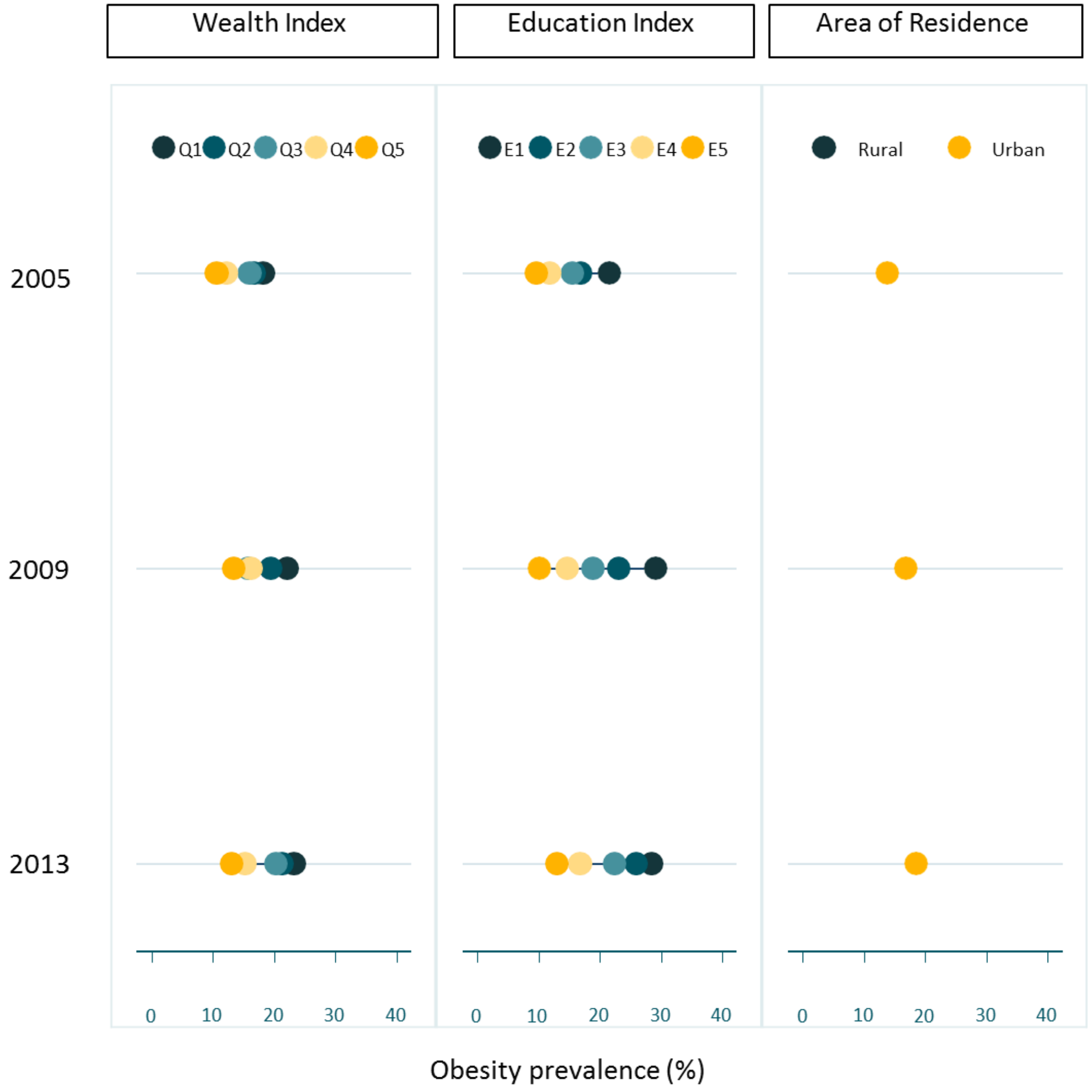

*only urban population sampled in Argentina

Supplementary figure 1B. Trend in age-standardized obesity prevalence by wealth, education and residence among adult men in Argentina*

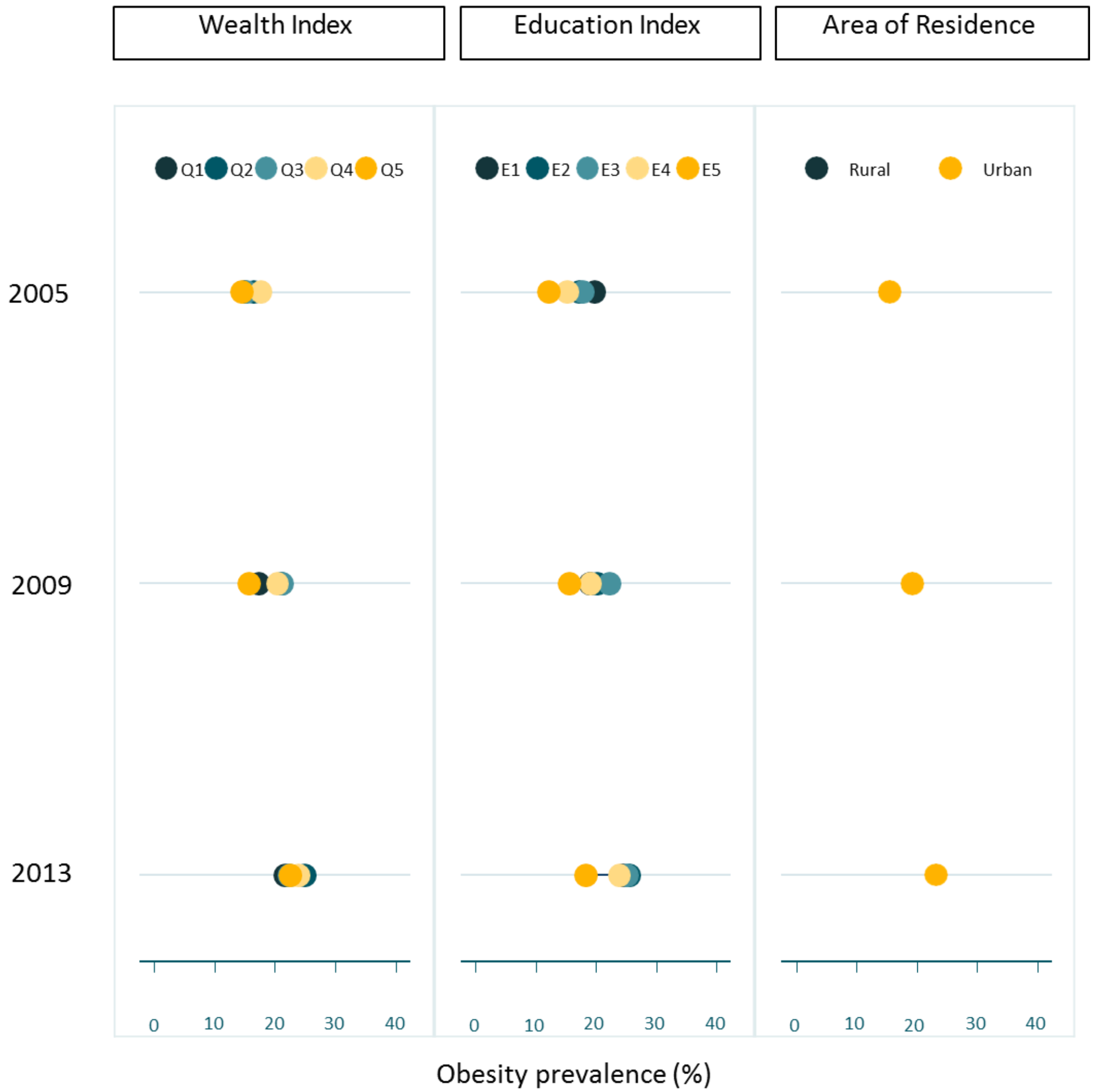

*only urban population sampled in Argentina

Supplementary figure 2A. Trend in age-standardized obesity prevalence by wealth, education and residence among adult women in Mexico

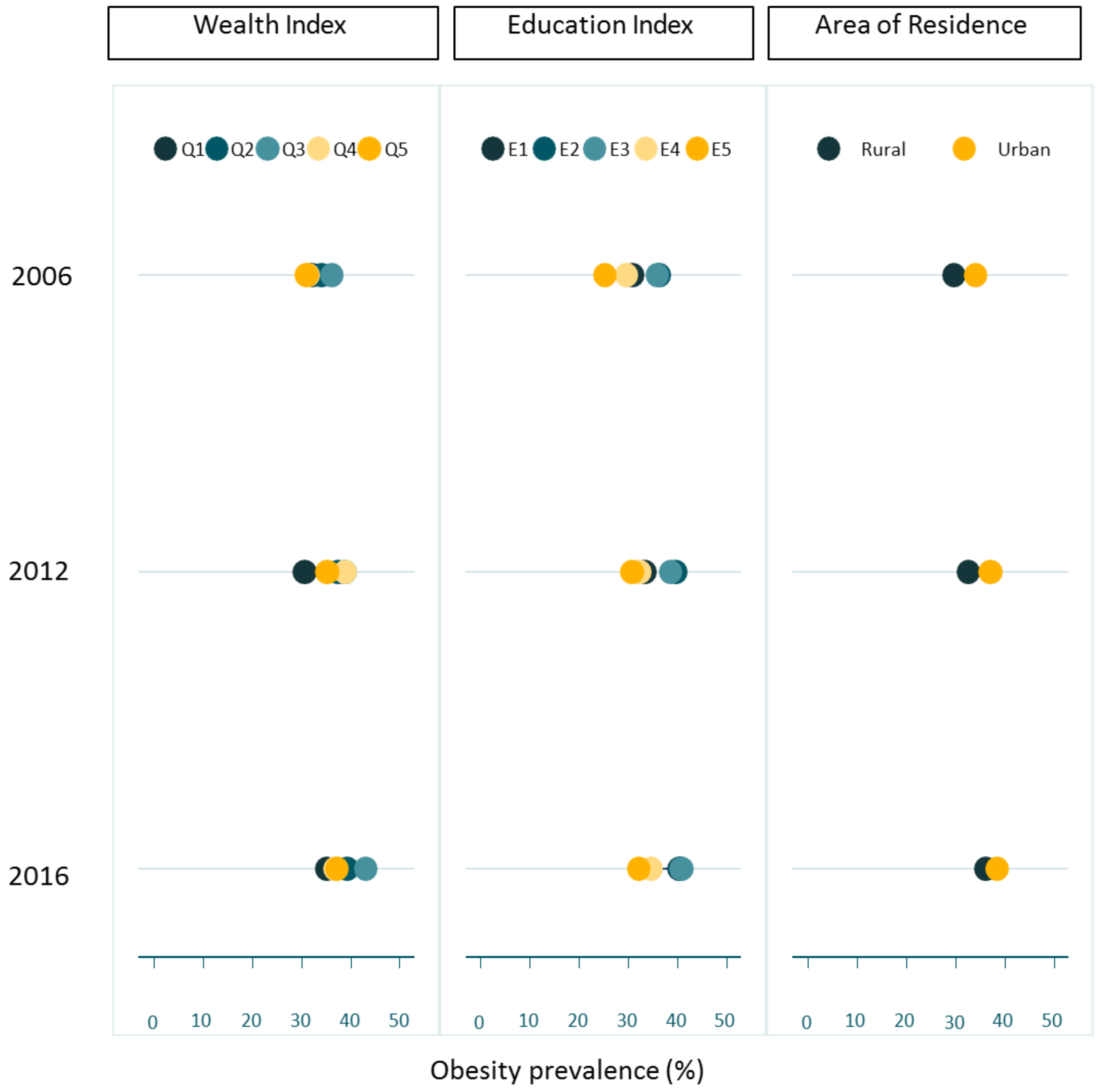

Supplementary figure 2B. Trend in age-standardized obesity prevalence by wealth, education and residence among adult men in Mexico

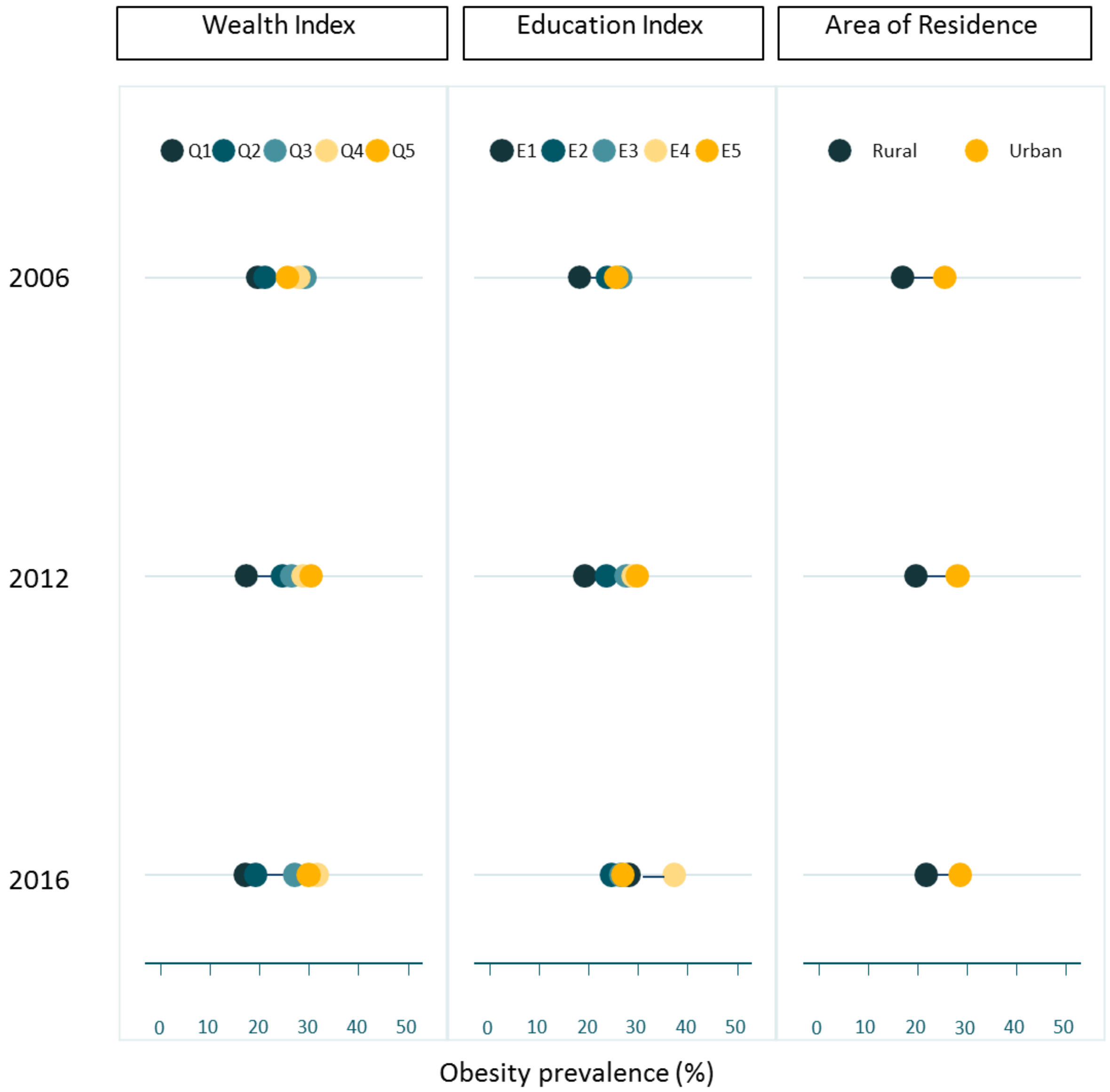

Supplementary figure 3A. Trend in age-standardized obesity prevalence by wealth, education and residence among adult women in Peru

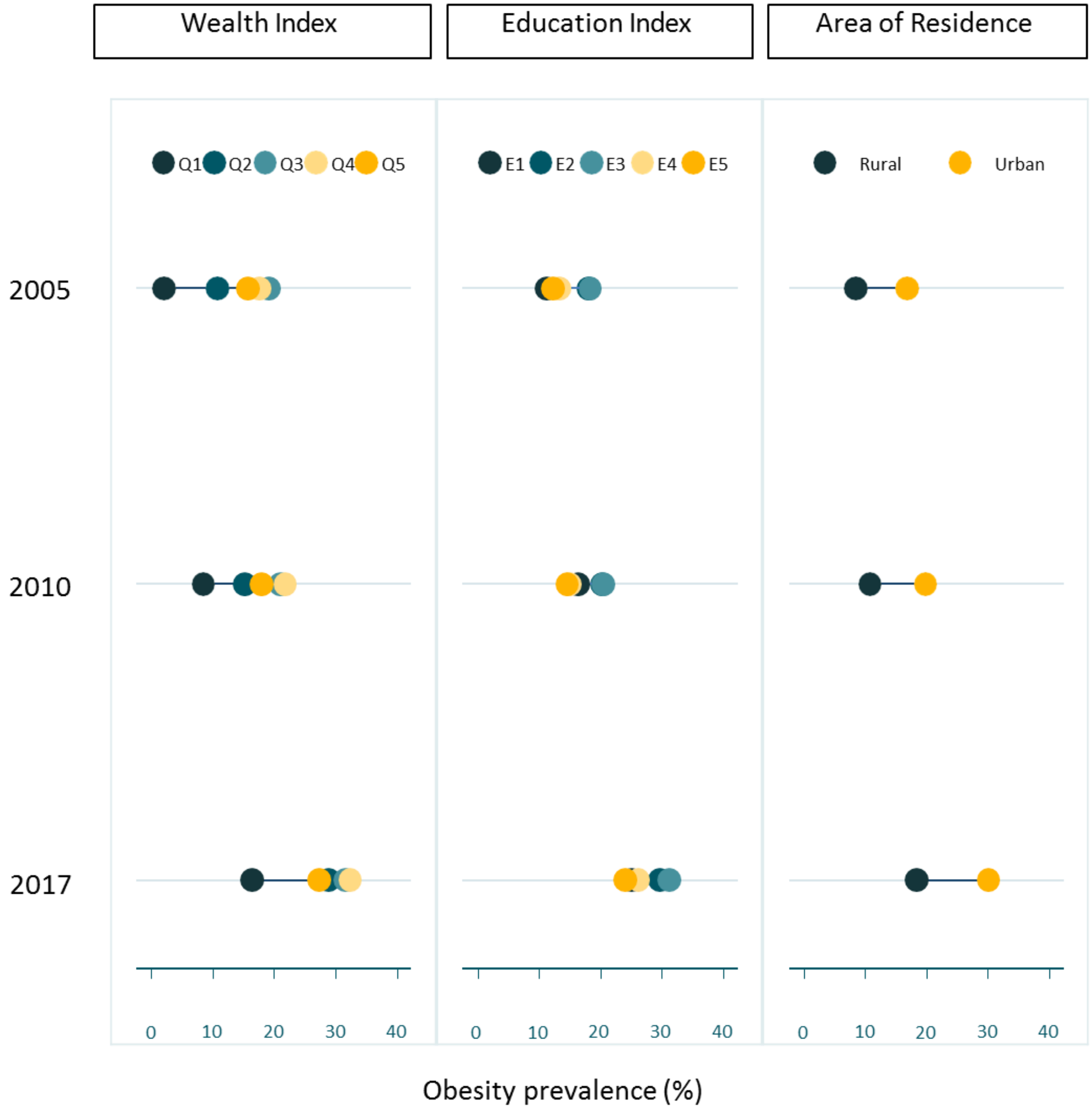

Supplementary figure 4A. Trend in age-standardized obesity prevalence by wealth, education and residence among adult women in Haiti

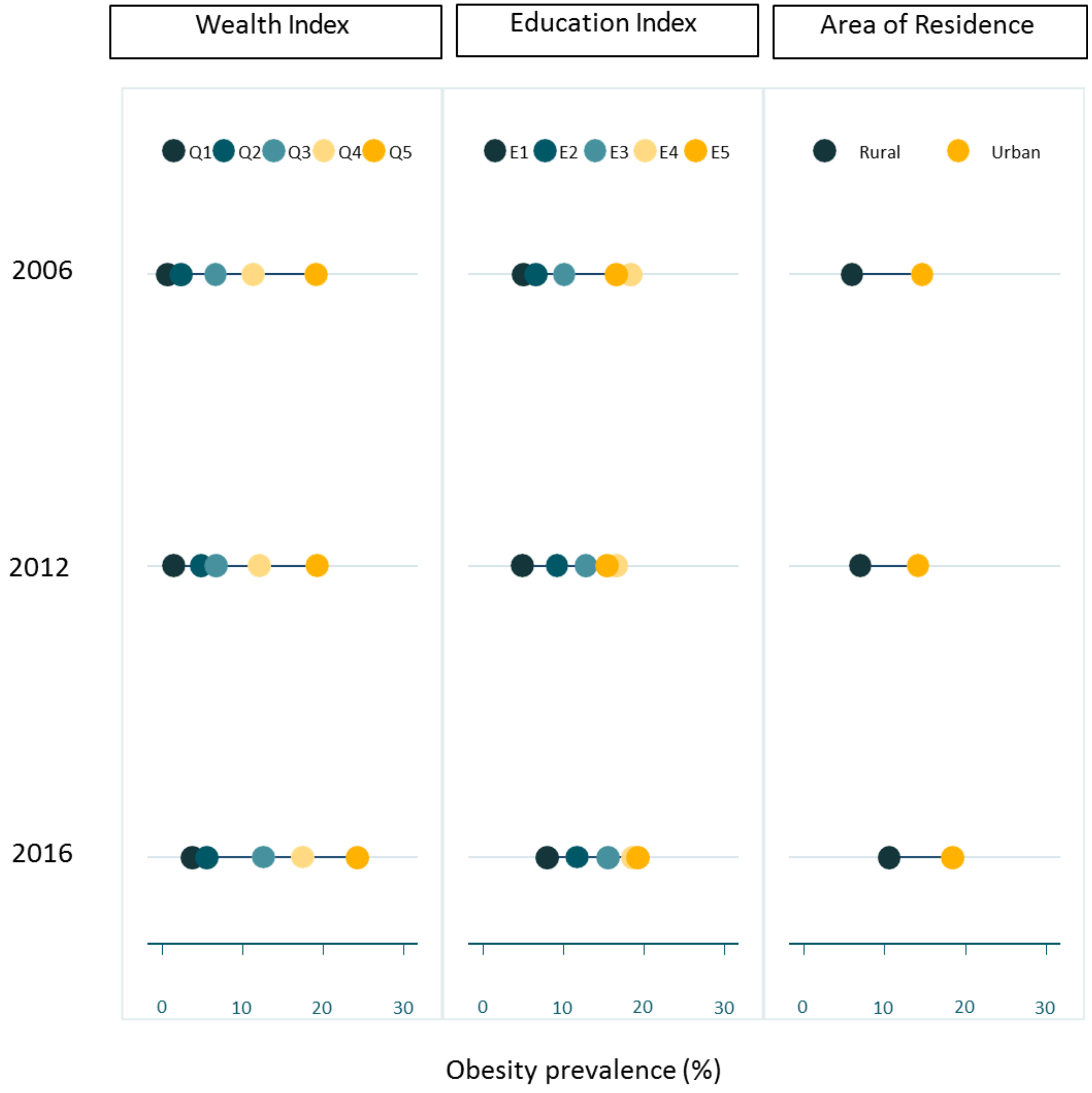

Supplementary figure 5A. Trend in age-standardized obesity prevalence by wealth, education and residence among adult women in Bolivia

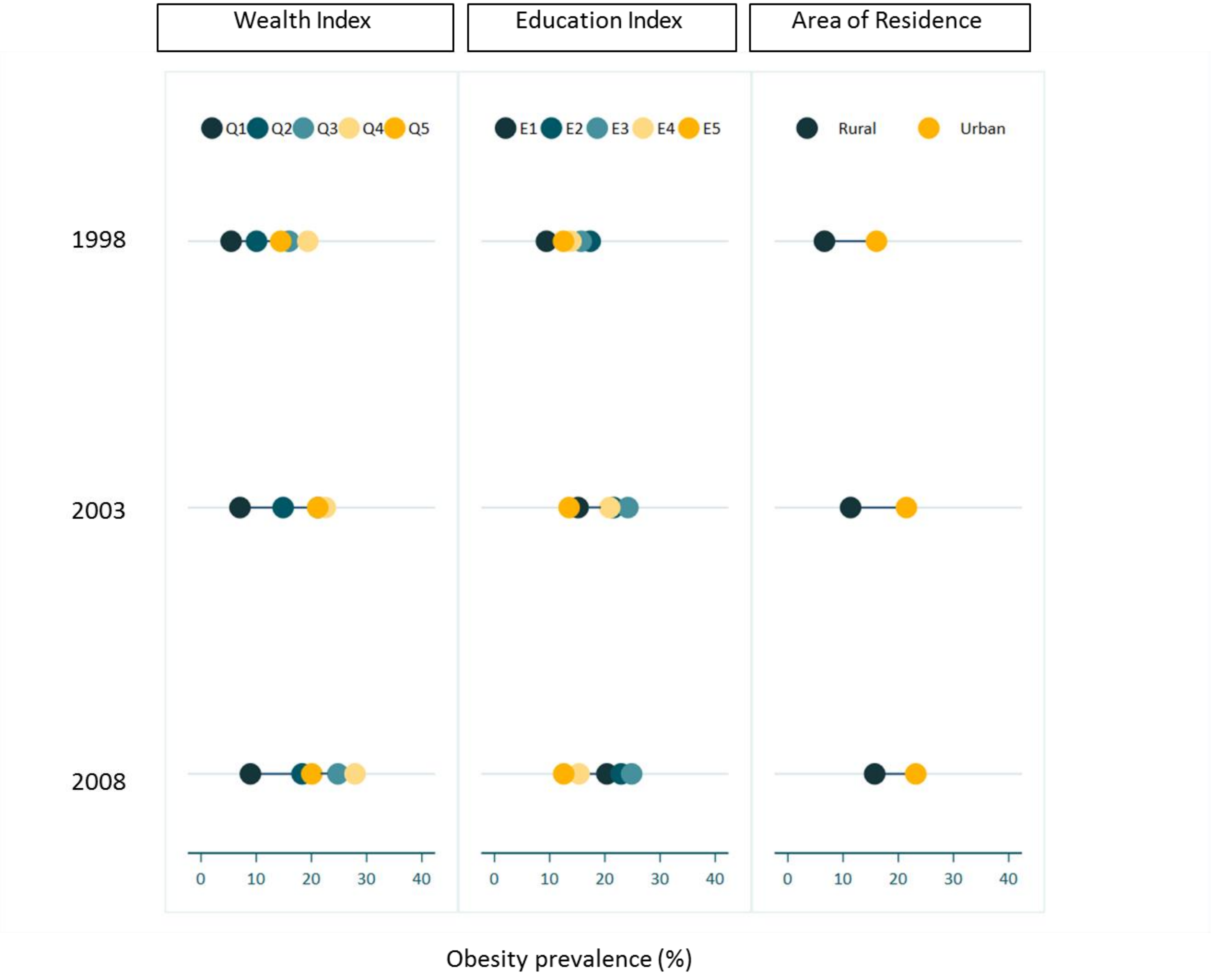

Supplement: Supplementary Materials [file EMS150063-supplement-Supplementary_Materials.zip › 1-s2.0-S2214109X19304218-mmc4.pdf]
