# Supplementary material for: The shift of obesity burden by socioeconomic status between 1998 and 2017 in Latin America and the Caribbean: a cross-sectional series study
Source: Lancet Glob Health. Author manuscript; Available in PMC 2022 Jul 17. (PMC7613084; doi:10.1016/S2214-109X(19)30421-8)
Supplement: Supplementary Materials [file EMS150063-supplement-Supplementary_Materials.zip › 1-s2.0-S2214109X19304218-mmc1.pdf]

THE LANCET
Global Health

Supplementary appendix 1

This translation in French was submitted by the authors and we reproduce it as supplied. It has not been peer reviewed. *The Lancet Global Health*'s editorial processes have only been applied to the original in English, which should serve as reference for this manuscript.

Supplement to: Jiwani SS, Carrillo-Larco RM, Hernández-Vásquez A, et al. The shift of obesity burden by socioeconomic status between 1998 and 2017 in Latin America and the Caribbean: a cross-sectional series study. *Lancet Glob Health* 2019; **7:** e1644–54.

Traduction

Cette traduction en français a été proposée par les auteurs et nous l'avons reproduite telle quelle. Elle n'a pas été examinée par des pairs. Les processus éditoriaux de *Lancet Global Health* n'ont été appliqués qu'à l'original en anglais, ce qui devrait servir de référence à ce manuscrit.

Supplement to: Jiwani SS, Carrillo-Larco RM, Hernández-Vásquez A, et al. Déplacement du fardeau de l'obésité selon le statut socioéconomique entre 1998 et 2017 en Amérique latine et dans les Caraïbes: une étude en série transversale. *Lancet Glob Health* 2019; **7:** e1644–54.

Sommaire

Contexte: Le fardeau de l'obésité diffère selon le statut socio-économique. Notre objectif est de caractériser la prévalence de l'obésité chez les hommes et les femmes adultes en Amérique latine et dans les Caraïbes selon des mesures socio-économiques et l'évolution du fardeau de l'obésité au fil du temps.

Methodes: Nous avons fait une analyse chronologique transversale de la prévalence de l'obésité en fonction du statut socio-économique à l'aide d'enquêtes nationales sur la santé menées entre 1998 et 2017 dans 13 pays d'Amérique latine et des Caraïbes. Nous avons généré des equiplots pour afficher les inégalités en matière de notre résultat primaire, l'obésité, selon la richesse, l'éducation et le milieu de résidence. Nous avons mesuré les écarts d'obésité définis comme étant la différence en points de pourcentage entre la prévalence d'obésité la plus élevée et la plus faible au sein de chaque mesure socio-économique, et nous avons décrit les tendances ainsi que les modèles changeants du fardeau de l'obésité au fil du temps.

Résultats: 479.809 hommes et femmes adultes ont été inclus dans l'analyse. La prévalence de l'obésité a augmenté à travers les pays, avec des modèles distincts apparaissant selon l'indice de richesse et d'éducation. Dans les enquêtes les plus récentes, l'obésité était plus prévalente parmi les femmes au Mexique en 2016 et moins prévalente parmi les femmes en Haïti en 2016. L'écart le plus important entre les estimations d'obésité les plus élevées et les moins élevées selon le niveau de richesse a été observé au Honduras chez les femmes (21,6 points de pourcentage), et au Pérou chez les hommes (écart de 22,4 points de pourcentage), par rapport à un écart de 3,7 points de pourcentage parmi les femmes au Brésil et 3,3 points de pourcentage parmi les hommes en Argentine. Les citadins avaient un fardeau plus lourd que leurs homologues ruraux dans la majorité des pays, avec un écart d'obésité allant de 0,1 points de pourcentage chez les femmes au Paraguay à 15,8 points de pourcentage parmi les hommes au Pérou. L'analyse des tendances réalisée dans cinq pays suggère un déplacement de la charge de l'obésité parmi les groupes socio-économiques et des modèles différents selon le sexe. Les écarts d'obésité selon l'éducation au Mexique se sont réduits au fil du temps parmi les femmes mais se sont accrus chez les hommes, tandis que l'écart s'est accru parmi les femmes mais reste relativement constant parmi les hommes en Argentine.

Interprétation: La hausse de la prévalence de l'obésité dans la région de l'Amérique latine et des Caraïbes s'est accompagnée d'une répartition inégale et d'un fardeau changeant parmi les groupes socio-économiques. L'anticipation de l'établissement de l'obésité parmi les groupes à faible niveau socio-économique fournit des opportunités de progrès pour la société en matière de prévention primordiale.
