# Supplementary material for: The shift of obesity burden by socioeconomic status between 1998 and 2017 in Latin America and the Caribbean: a cross-sectional series study
Source: Lancet Glob Health. Author manuscript; Available in PMC 2022 Jul 17. (PMC7613084; doi:10.1016/S2214-109X(19)30421-8)
Supplement: Supplementary Materials [file EMS150063-supplement-Supplementary_Materials.zip › 1-s2.0-S2214109X19304218-mmc2.pdf]

THE LANCET
Global Health

Supplementary appendix 2

This translation in Portuguese was submitted by the authors and we reproduce it as supplied. It has not been peer reviewed. *The Lancet Global Health*'s editorial processes have only been applied to the original in English, which should serve as reference for this manuscript.

Supplement to: Jiwani SS, Carrillo-Larco RM, Hernández-Vásquez A, et al. The shift of obesity burden by socioeconomic status between 1998 and 2017 in Latin America and the Caribbean: a cross-sectional series study. *Lancet Glob Health* 2019; **7:** e1644–54.

Tradução

Esta tradução em Português foi submetida pelos autores e a reproduzimos aqui como foi fornecida. Não foi revista por pares. Os processos editoriais da *Lancet Global Health* só foram aplicados ao original em inglês, e este deve servir de referência para este manuscrito.

Supplement to: Jiwani SS, Carrillo-Larco RM, Hernández-Vásquez A, et al. A transição na carga de obesidade segundo o nível socioeconômico entre 1998 e 2017 na América Latina e no Caribe: estudos transversais seriados. *Lancet Glob Health* 2019; **7:** e1644–54.

RESUMO

Antecedentes: A carga representada pela obesidade varia de acordo com o nível socioeconômico. O objetivo do estudo foi mensurar a prevalência de obesidade entre adultos (homens e mulheres) na América Latina e no Caribe segundo medidas socioeconômicas e a tendência temporal na ocorrência da obesidade.

Métodos: Série de estudos transversais da prevalência de obesidade segundo nível socioeconômico, utilizando pesquisas nacionais de saúde realizadas entre 1998 e 2017 em 13 países da América Latina e Caribe. Foram gerados *equiplots* para evidenciar desigualdades na obesidade segundo riqueza (posse de bens), escolaridade e local de residência. Foram mensuradas as desigualdades na obesidade - diferença em pontos percentuais entre a maior e a menor prevalência de obesidade dentro de cada indicador socioeconômico - e descritas as tendências– mudanças no padrão da carga de obesidade ao longo do tempo.

Resultados: 479.809 indivíduos foram incluídos na análise. As prevalências de obesidade segundo os países aumentaram ao longo do tempo, com padrões distintos segundo riqueza e escolaridade. Nas pesquisas mais recentes, a obesidade foi mais prevalente entre as mulheres no México em 2016 e menos prevalente entre as mulheres no Haiti em 2016. A maior diferença na obesidade segundo riqueza foi observada em Honduras entre as mulheres (21,6 pontos percentuais - pp) e no Peru entre os homens (22,4 pp), comparado com uma diferença de 3,7pp entre as mulheres no Brasil e 3,3 pp entre os homens na Argentina. Residentes na zona urbana apresentaram, consistentemente, uma maior carga de obesidade do que residentes na área rural na maioria dos países, com diferenças variando de 0,1 pp entre as mulheres no Paraguai a 15,8 pp entre os homens no Peru. A análise de tendência conduzida em cinco países sugere uma mudança da carga de obesidade entre grupos socioeconômicos e diferentes padrões por gênero. No México, a diferença na ocorrência de obesidade segundo escolaridade reduziu ao longo do tempo entre as mulheres, mas aumentou entre os homens. Na Argentina, a diferença aumentou entre as mulheres, mas permaneceu relativamente constante entre os homens.

Interpretação: O aumento da obesidade na região da América Latina e do Caribe foi desigual entre os grupos socioeconômicos. Esforços para prevenir a ocorrência da obesidade entre os grupos de menor nível socioeconômico podem oportunizar maior efetividade da prevenção primordial.
