# Supplementary material for: The shift of obesity burden by socioeconomic status between 1998 and 2017 in Latin America and the Caribbean: a cross-sectional series study
Source: Lancet Glob Health. Author manuscript; Available in PMC 2022 Jul 17. (PMC7613084; doi:10.1016/S2214-109X(19)30421-8)
Supplement: Supplementary Materials [file EMS150063-supplement-Supplementary_Materials.zip › 1-s2.0-S2214109X19304218-mmc3.pdf]

THE LANCET
Global Health

Supplementary appendix 3

This translation in Spanish was submitted by the authors and we reproduce it as supplied. It has not been peer reviewed. *The Lancet Global Health*'s editorial processes have only been applied to the original in English, which should serve as reference for this manuscript.

Supplement to: Jiwani SS, Carrillo-Larco RM, Hernández-Vásquez A, et al. The shift of obesity burden by socioeconomic status between 1998 and 2017 in Latin America and the Caribbean: a cross-sectional series study. *Lancet Glob Health* 2019; **7:** e1644–54.

Traducción

Los autores nos proporcionaron esta traducción al español y la reproducimos tal como nos fue entregada. No la hemos revisado. Los procesos editoriales de *The Lancet Global Health* se han aplicado únicamente al original en inglés, que debe servir de referencia para este manuscrito.

Supplement to: Jiwani SS, Carrillo-Larco RM, Hernández-Vásquez A, et al. El cambio en la carga de obesidad por estado socioeconómico entre 1998 y 2017 en America Latina y el Caribe: un estudio de series transversales. *Lancet Glob Health* 2019; **7:** e1644–54.

RESUMEN

Antecedentes: La carga de obesidad varía según el estado socioeconómico. Nuestro objetivo fue caracterizar la prevalencia de obesidad en hombres y mujeres adultos en América Latina y el Caribe según indicadores socioeconómicos, así como el cambio en la carga de obesidad en el tiempo.

Métodos: Se realizó un análisis de datos de corte transversal de las prevalencias de obesidad según nivel socioeconómico utilizando encuestas nacionales de salud realizadas entre 1998 y 2017 en 13 países de América Latina y el Caribe. Mediante equiplots se identificaron las desigualdades en la prevalencia de obesidad según el índice de riqueza, niveles de educación y área de residencia. Se determinó las brechas de obesidad como la diferencia en puntos porcentuales entre la prevalencia de obesidad más alta y más baja de cada medida socioeconómica, y sus tendencias en el tiempo.

Resultados: Se incluyeron en el análisis 479 809 hombres y mujeres adultos. La prevalencia de obesidad ha aumentado en el tiempo, con distintos patrones según el índice de riqueza y niveles de educación. En las encuestas más recientes, la mayor prevalencia de obesidad se observó en las mujeres de México en 2016, y la menor en las mujeres de Haití en 2016. La mayor brecha entre las prevalencias de obesidad más altas y más bajas según el índice de riqueza se observó en las mujeres de Honduras (brecha de 21,6 puntos porcentuales), y en hombres de Perú (brecha de 22,4 puntos porcentuales), en comparación con una brecha de 3,7 puntos porcentuales en mujeres de Brasil y 3,3 puntos porcentuales en hombres de Argentina. Los residentes en áreas urbanas tuvieron una mayor prevalencia de obesidad que sus contrapartes del área rural en la mayoría de los países, con brechas que van desde 0,1 puntos porcentuales en las mujeres de Paraguay hasta 15,8 puntos porcentuales en los hombres de Perú. El análisis de tendencias llevado a cabo en cinco países sugiere un cambio en la carga de obesidad de acuerdo a los grupos socioeconómicos y diferentes patrones por género. En México, las brechas de obesidad en el tiempo según el nivel educativo disminuyeron con el tiempo en las mujeres pero aumentaron en los hombres, mientras que en Argentina la brecha aumentó en las mujeres pero se mantuvo relativamente constante entre los hombres.

Interpretación: El incremento en la prevalencia de obesidad en la región de América Latina y el Caribe muestra una distribución desigual y una transición entre los grupos socioeconómicos. Anticipar el desarrollo de la obesidad en los grupos socioeconómicos bajos genera oportunidades para ganancias sociales en prevención primordial.
